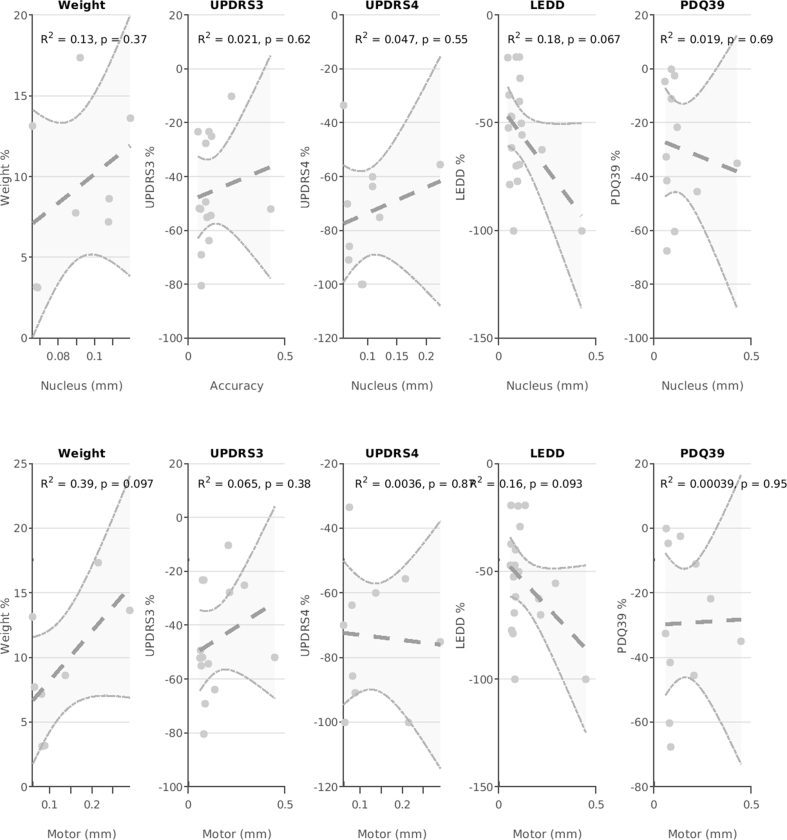# Increased variance in second electrode accuracy during deep brain stimulation and its relationship to pneumocephalus, brain shift, and clinical outcomes: A retrospective cohort study

**DOI:** 10.1016/j.bas.2022.100893

**Published:** 2022-05-21

**Authors:** M.G. Hart, M. Posa, P.C. Buttery, R.C. Morris

**Affiliations:** a St George’s University of London, Cranmer Terrace, London, SW17 0RE, UK; b Department of Neurosurgery, Addenbrooke’s Hospital, Cambridge, CB2 0QQ, UK; c Department of Neurology, Addenbrooke’s Hospital, Cambridge, CB2 0QQ, UK

**Keywords:** Globus pallidus internus, Magnetic resonance imaging, Parkinson’s disease, Subthalamic nucleus

## Abstract

• Overall electrode accuracy was 0.22+/-0.4 ​mm with only 3 (4%) electrodes out with 2 ​mm from the intended target.
• Accuracy was significantly worse in the GPi versus the STN and on the second side implanted.
• Inaccuracy occurred in the X (lateral) plane but was not related to pneumocephalus or brain shift.

## 1 Introduction

Deep brain stimulation for Parkinson’s disease is an effective treatment supported by evidence from randomised controlled trials(Deuschl et al., 2006; Weaver et al., 2009; Williams et al., 2010; Okun, 2012; Schuepbach et al., 2013) and is now established in routine clinical practice internationally. Despite the ubiquity and success of the procedure, methods for appraisal of electrode accuracy – a key surgical outcome variable(Saint-Cyr et al., 2002; McClelland et al., 2005; III et al., 2007; Pilitsis et al., 2008) have not yet been formalised. Failure to appreciate electrode accuracy may lead to excessive revision rates, unsatisfactory clinical outcomes, and an inability to effectively appraise surgical results.

Placement of electrodes has historically relied on microelectrode recordings (MER) and intraoperative macrostimulation during awake surgery(Bari et al., 2018). More recently, image-guided surgery under general anaesthesia without MER has been developed for reasons of efficiency, patient comfort, and safety(Burchiel et al., 2013; Chen et al., 2017; Ho et al., 2018; Ko and Burchiel, 2018). Methods for appraising electrode accuracy with either technique are usually based on imaging, and while commercial and open-source software is available for this purpose(Miocinovic et al., 2007; D’ Albis et al., 2015; Horn and K ü hn, 2015; Silva et al., 2015; Lauro et al., 2016; Husch et al., 2018; Horn et al., 2019a), reports of applications of these methods in routine clinical practice are few.

Our aim was to test the applicability of image-based electrode localisation, specifically using the open-source Lead-DBS toolbox(Horn and K ü hn, 2015; Horn et al., 2019a), in routine clinical practice. In particular, we wished to test the ability to interrogate the accuracy of our own deep brain stimulation practice for Parkinson’s disease using a direct targeting, MRI guided, and CT verified technique under general anaesthesia. We hypothesised that the volume of the target nucleus that was stimulated would correspond most strongly with motor outcomes. Furthermore, we hypothesised that inaccuracy would be related to well-known variables, namely pneumocephalus, intraoperative brain shift, and whether the electrode was inserted first or second.

## 2 Materials and methods

### 2.1 Patients

A retrospective cohort study was performed of a consecutive series of patients with Parkinson’s disease who underwent deep brain stimulation of either the GPi or STN between 2016 and 2019. Patient selection for deep brain stimulation was performed in a multi-disciplinary setting according to national commissioning criteria(Board, 2013; (NICE) NI for H and CE, 2017). Targeting of the subthalamic nucleus (STN) or globus pallidus internus (GPi) was based on individual treatment goals as part if a multidisciplinary team assessment. Baseline patient details are presented in Table 1. Institutional ethical approval was granted as a review of service study.

**Table 1:** Cohort baseline clinical and demographic data.

Feature		Value
Gender	Female	14
	Male	24
Age		62.1 (7.8)
Disease duration		11.8 (6.7)
Target	GPi	15
	STN	23
Manufacturer	Abbott/St Jude	28
	Boston Scientific	7
	Medtronic	3
UPDRS 3		48.1 (9.5)
UPDRS 4		8.6 (4.4)
LEDD		1112.8 (575.3)
PDQ39		76.5 (22.5)
MOCA		25.8 (4.1)

All results for UPDRS are off medication. Continuous measures are mean (+/- 2 standard deviation). Time data is in years. Levodopa equivalent daily dose (LEDD) is in mg.

### 2.2 Surgical procedure

Surgery was performed as a single stage procedure under general anaesthesia with implantation of electrodes manufactured by: St Jude/Abbott (Abbott Laboratories, Lake Bluff, Illinois, USA: 6147 non-directional electrode with Libra PC system, or 6170 directional electrode with Infinity system), Medtronic (Medtronic Inc, Dublin, Eire: 3389 electrode with Activa PC system), or; Boston (Boston Scientific, Marlborough, Mass, USA: Cartesia directional electrode and Vercise PC or Gevia system). Planning was performed using StealthStation® S7^TM^ (Medtronic Inc, Dublin, Eire) FrameLink® software. Direct targeting was performed based on pre-operative 3 ​T MRI data (magnetization-prepared rapid-acquisition gradient-echo (MPRAGE) volumetric STEALTH sequences, proton density (PD) sequences for the GPi, and susceptibility-weighted imaging (SWI) sequences for the STN. Targets were planned to be based in the centre of the motor component of the nucleus. A Leksell frame (Elekta AB, Stockholm, Sweden) was used in combination with pre-operative and post-operative volumetric CT imaging for trajectory planning and verification, respectively. If post-operative CT imaging demonstrated satisfactory appearances the implantable pulse generator was placed during the same general anaesthetic. In all cases the left hemisphere was implanted first.

### 2.3 Image registration, electrode reconstruction, and calculation of Volume of Activated Tissue (VAT)

**Fig. 1:**
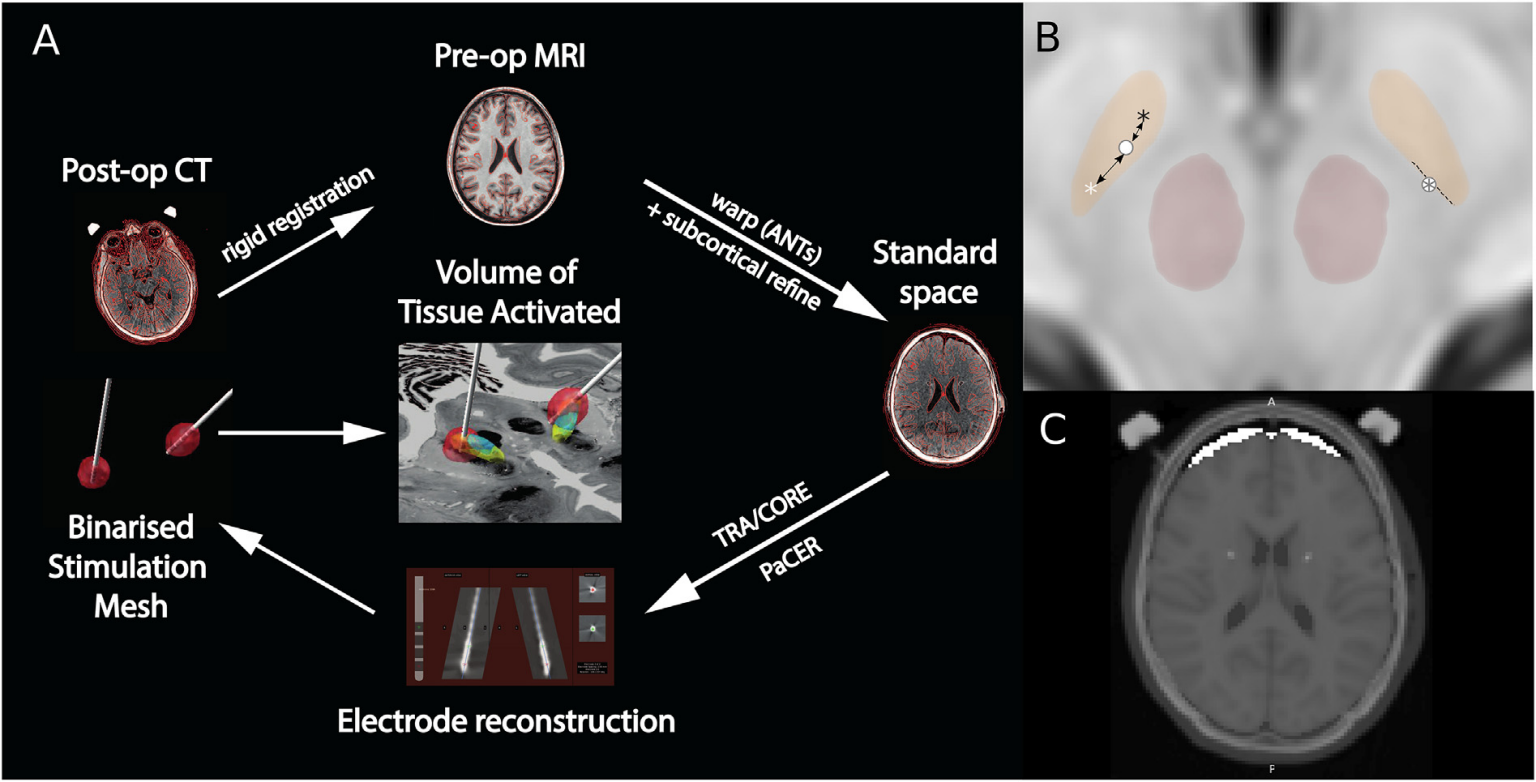
Image Processing Pipeline A: Post-operative CT scans were linearly registered to the pre-operative MRI which in turn was registered to the standard space of the Montreal Neurological Institute using non-linear diffeomorphic warping. Subsequently, subcortical structures were subject to an additional rigid registration to account for brain shift (subcortical refine). Both transforms were combined and applied. Electrode reconstruction was performed using either PaCER for non-directional leads or TRA/CORE for directional leads. Subsequently a Volume of Activated Tissue (VAT) was generated as a mesh and displayed on a template atlas. B: electrode distances were defined according to distance from nucleus border (right) for analysis of accuracy, or distance to either the main nucleus (grey asterisk) or motor subnucleus (white asterisk) when analysing XYZ variance.

Image processing was performed using the Lead-DBS toolbox (Horn and K ü hn, 2015; Horn et al., 2019a) (Fig. 1A). Registration of the post-operative CT image to the standard space template of the Montreal Neurological Institute (MNI152) was performed using Advanced Normalization Tools (ANTs) (Avants et al., 2009; Tustison et al., 2013). Specifically, post-operative CT images were linearly registered to the pre-operative T1-weighted MPRAGE image which in turn was registered to the ICBM152 2009b template with non-linear diffeomorphic warping. Brain shift correction of subcortical structures was performed using linear registration of an additional subcortical mask(Sch ö necker et al., 2009).

Electrodes were reconstructed based on the post-operative CT images using a combination of PaCER (Husch et al., 2018) algorithm or if this failed TRA/CORE (Horn and K ü hn, 2015; Horn et al., 2019a). Subsequently manual refinement was performed to align the electrode model with the imaging artefact on the corresponding CT image.

Volume of Activated Tissue (VAT) reconstructions were performed using a finite element model implemented in Lead-DBS(Horn and K ü hn, 2015; Horn et al., 2019a). A tetrahedral mesh was constructed based on a four-compartment model comprising grey matter, white matter, and electrode (conducting and non-conducing) components. Conductivity values were set according to standard parameters then the VAT was binarised at a threshold of 0.2 ​V/mm. Finally, electrode locations and VATs were visualised on the DISTAL atlas(Ewert et al., 2018).

### 2.4 Definition of accuracy

Electrode accuracy was defined as the shortest distance between any electrode contact and the boundary of the target region (either main nucleus or motor subnucleus) (Fig. 1B). This was defined in both the 2D plane for target plots and in terms of 3D Euclidean distance. Electrode variance was defined as the distance in 3D Euclidean co-ordinates from the centre of gravity of the target in question (either the main nucleus of motor subnucleus).

### 2.5 Computation of brain shift and pneumocephalus

Brain shift is believed to occur after durotomy and results in a complex and likely nonlinear displacement of the brain that is poorly defined. This can result in significant difficulties in registration between images as nonlinear transforms can impact on electrode localisation robustness. Subsequently, one of the most well-developed methods to account for this relies upon using layered linear transforms to subcortical regions of interest ((Sch ö necker et al., 2009)). We quantified this approach by summing the resultant transformation matrix. Additionally, we localised electrodes both with and without this approach to assess the difference it made to accuracy. Pneumocephalus is typically related to durotomy and believed to be associated with brain shift. We quantified the degree of pneumocephalus by using an MNI space brain mask which defined pneumocephalus as the difference between this as the expected brain volume and the actual extracted brain volume (Fig. 1C).

### 2.6 Outcome assessment

Programming commenced at approximately 6 weeks following surgery and was led by a consultant neurologist with a specialist interest in movement disorders in combination with a specialist nurse. Initial programming commenced with a pulse width of 60μs and a frequency of 130Hz. Outcome variables were recorded by a consultant neurologist in combination with a specialist nurse at 12 months following surgery. Outcome measures included: body weight (in kilograms); UPDRS 3; UPDRS 4; levodopa equivalent daily dose (LEDD); and PDQ39.

### 2.7 Statistical analysis

Groups were analysed according to hemisphere (with the second electrode to be implanted being in the right hemisphere), nucleus (GPi or STN), and target (main nucleus or motor subnucleus). Distances from the electrode to, and overlap of the VAT with, the corresponding nucleus and specific component of the nucleus were calculated. All values are expressed as mean+/-2 standard deviations (SD). Raincloud plots were generated to display raw data, box plots, and half-violin plots of the data distribution ((Allen et al., 2019)).

Differences in continuous variables were performed with paired t-tests (dual groups) or Analysis of Variance (ANOVA, multiple groups). Statistical dependencies between continuous variables were analysed with Pearson’s correlation. A general linear model was fitted on clinical outcomes, electrode accuracy, and VAT’s. Significance was set at p<0.05 with corrections for multiple comparisons using the Bonferroni method. All analyses were performed in MATLAB (version 9.7.0 (R2021a), Natick, Massachusetts: The MathWorks Inc.) using open-source code (https://github.com/jazzmanmike/DBS/).

## 3 Results

### 3.1 Cohort characteristics

In total 38 participants met the inclusion criteria. Baseline clinical and demographic data of the cohort are presented in Table 1. The only clinical adverse events were a device infection that required system explantation and tethering of an implantable pulse generator managed with revision surgery.

### 3.2 Image Processing

Overall, 30 out of 38 (79%) participants successfully completed the image processing pipeline (Supplementary Fig. 1). Reasons for exclusion from analysis included registration failure (post-op CT to pre-op MRI, 6 participants) and failure of VAT construction (2 participants). Correction for brain shift with subcortical refine methodology was utilised in all cases. Electrode reconstructions were performed primarily with PaCER, or TRA/CORE if this was not possible (15 participants each).

### 3.3 Clinical outcomes

**Fig. 2:**
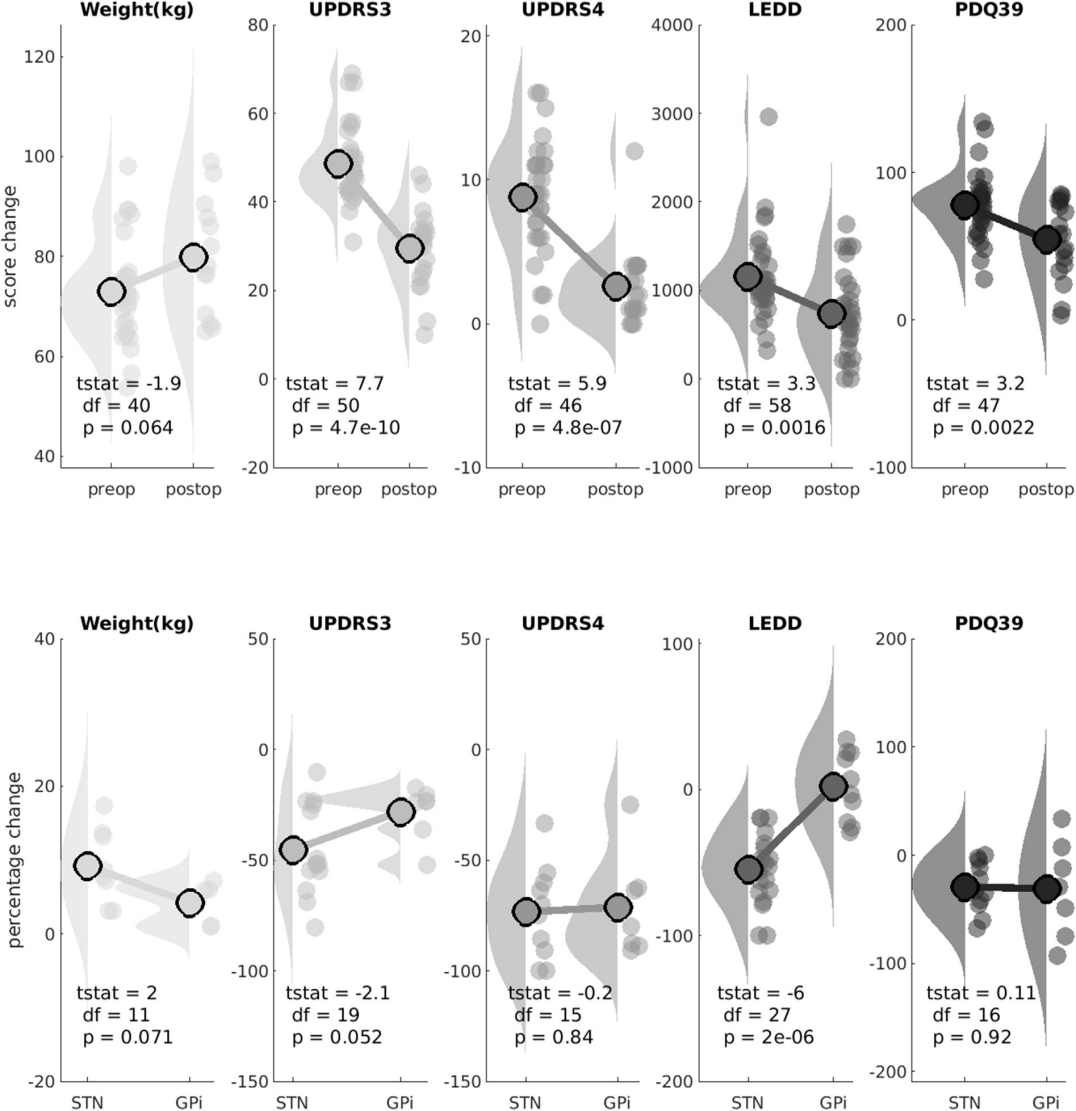
Clinical Outcomes Raincloud plots of changes in the five clinical outcome variables. Upper rows (green) are pre-operative values, lower rows (orange) are post-operative scores. All changes are as percentages. tstat ​= ​paired *t*-test value. Raincloud plots display the raw data, box plots, and half-violin plots of the data distribution ((Allen et al., 2019)).

Changes in clinical outcomes post-operatively are presented in Fig. 2 and Table 2. In the overall cohort, statistically significant improvements were demonstrated for UPDRS 3, UPDRS 4, LEDD, and PDQ39. Stimulation of the STN compared with the GPi resulted in a significantly greater reduction in LEDD (55% decrease versus 2% increase respectively, p<0.001) but otherwise there were no differences in outcomes. Finally, all the described clinical outcomes were independent and did not demonstrate significant covariance (Supplementary Fig. 2: maximal R^2^ 0.13, p ​= ​0.12).

**Table 2:** Clinical outcomes.

	Overall	Target		Gender	
		GPi	STN	Female	Male
UPDRS 3	−45.9% (23.5)	−30.5% (13.8)	−51.8% (24.1)	−32.9% (13.3)	−54.2% (25.2)
UPDRS 4	−72.2% (23.5)	−76.5% (12.7)	−70.1% (44.7)	−77.0% (16.9)	−69.8% (44.0)
LEDD	−35.9% (36.1)	0.9% (25.1)	−54.3% (24.9)	−27.6% (33.2)	−41.8% (38.1)
PDQ39	−32.1% (34.6)	−31.1% (53.3)	−32.6% (24.4)	−31.5% (31.9)	−32.5% (38.1)
Weight	8.7% (5.4)	5.0% (2.7)	10.8% (5.6)	10.6% (5.9)	9.0% (5.5)

Motor scores are off medication. Continuous measures are mean (+/- 2 standard deviations). Time data is in years. LEDD is in mg.

### 3.4 electrode accuracy

**Fig. 3:**
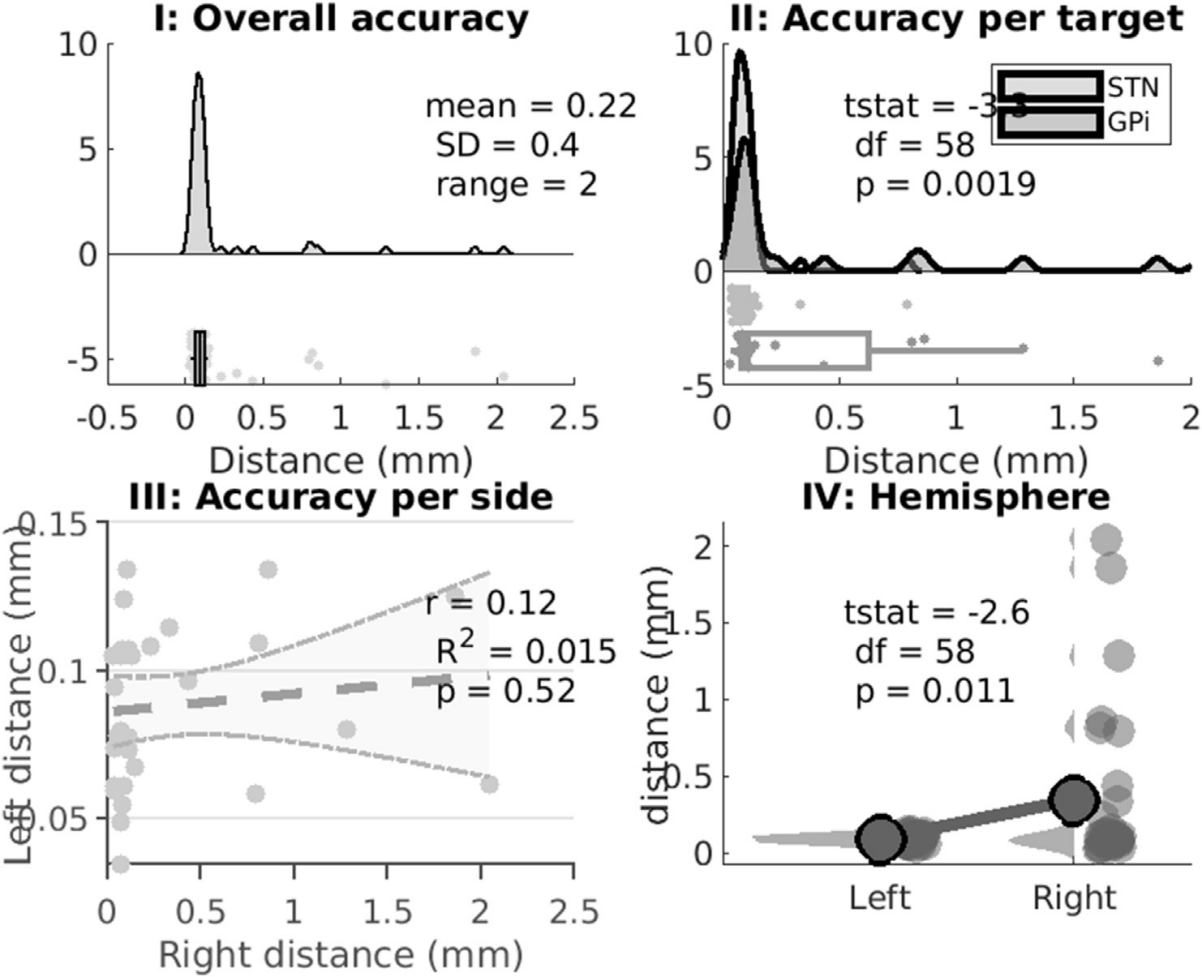
Electrode Accuracy Summary I: Overall accuracy for all electrodes for both targets (GPi and STN) and hemispheres (60 electrodes). In total 9 outliers were identified and only 3 electrodes out with 2 ​mm from the intended target. II: Electrodes implanted in the GPi had lower accuracy than those implanted in the STN. III: Electrode accuracy did not systemically vary between sides (i.e. it was not the case that low accuracy in one hemisphere was associated with low in the other hemisphere, related to for example a shared methodological step). IV: Electrode accuracy varied between hemispheres with reduced accuracy in the right hemisphere which was the second side implanted.

**Fig. 4:**
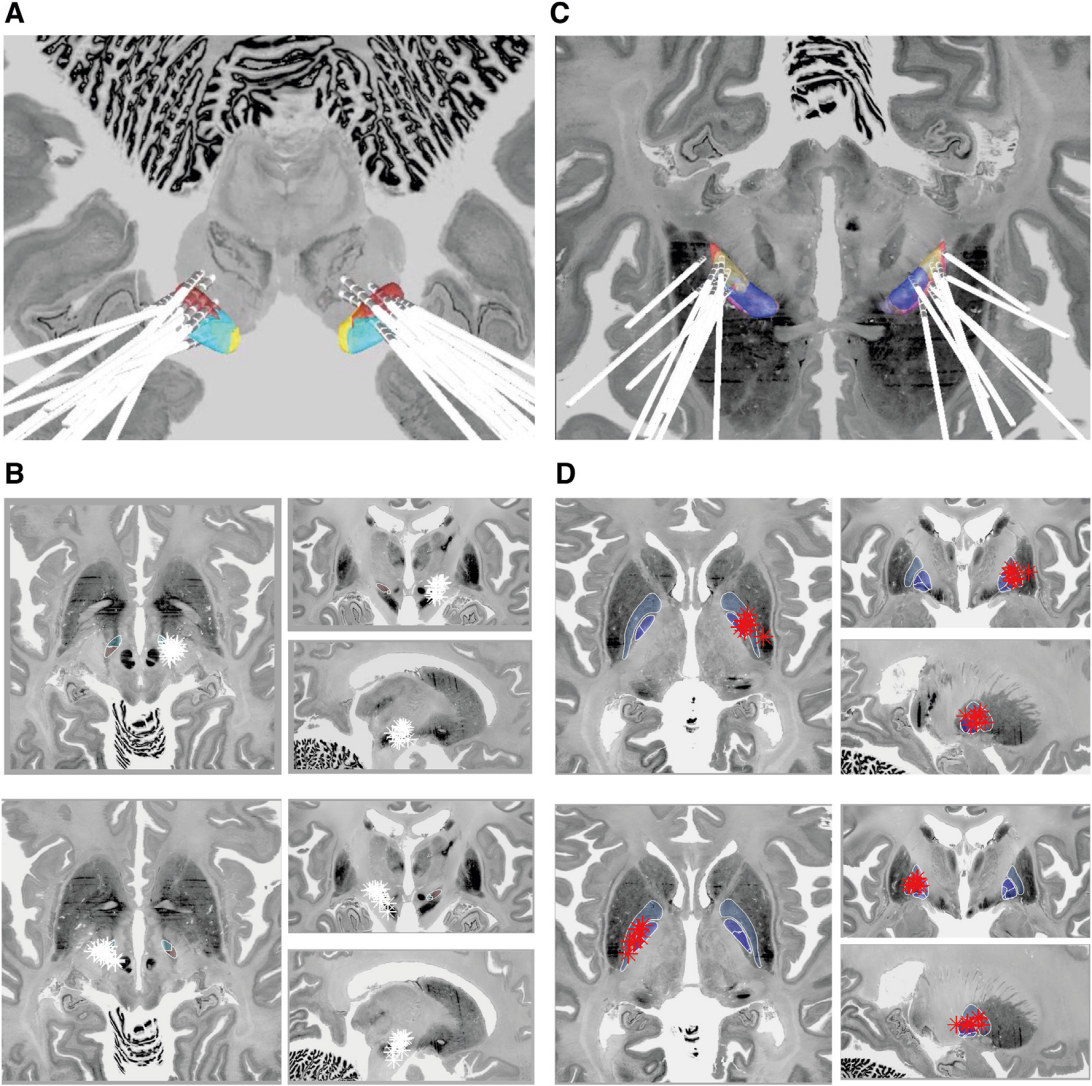
Group Electrode Location Visualisation A: subthalamic nucleus electrode reconstructions B: corresponding subthalamic nucleus electrode contacts on the right (upper) and left (lower). C: globus pallidus internus electrode reconstructions D: corresponding globus pallidus interna electrode contacts on the right (upper) and left (lower). The background image for all figures is from the BigBrain project (https://bigbrain.loris.ca/main.php?) under CC-BY-NC-SA 4.0 license.

Overall electrode accuracy was 0.22+/-0.4 ​mm for all electrodes to the main nucleus with 9 (12%) outliers but only 3 (4%) electrodes out with 2 ​mm from the intended target (Fig. 3, Fig. 4). For GPi stimulation, electrodes were a mean of 0.26+/-0.43 ​mm from the main nucleus, and 0.80+/-0.97 ​mm from the GPi motor nucleus. Overall, 17 out of 20 electrodes were within 2 ​mm of the main nucleus. For STN stimulation, electrodes were a mean of 0.14 ​mm (SD 0.28) from the main nucleus, and 0.27 ​mm (SD 0.52) from the STN motor nucleus. Overall, all 40 electrodes were within 2 ​mm of the main nucleus.

Nucleus (GPi or STN) affected accuracy with lower accuracy for electrodes in the GPi than the STN (0.43+/-0.62 ​mm versus 0.11+/-0.12 ​mm, p ​= ​0.002). There was no systematic co-variance in accuracy between hemispheres (r ​= ​0.12, R^2^ ​= ​0.01, p ​= ​0.52). However, the second electrode implanted (i.e. that in the right hemisphere) was less accurate than the first electrode implanted (0.09+/-0.03 ​mm versus 0.34+/-0.53 ​mm, p ​= ​0.01).

To determine which clinical scenario (GPi versus STN, main nucleus versus motor subnucleus, right versus left side) accuracy is most likely to be affected, a systematic analysis is presented in Supplementary Fig. 3. Concordant with the main findings above, variance in accuracy was most prominent in the right GPi (group F-stat ​= ​9.68, df ​= ​7, p ​< ​0.01; GPi right tstat ​= ​-2.9, df ​= ​18, p ​= ​0.01). Accuracy did not vary depending on whether the target reference was chosen to be the main nucleus or motor sub-nucleus (tstat ​= ​-1.1, df ​= ​118, p ​= ​0.27).

### 3.5 Systematic variance in accuracy

**Fig. 5:**
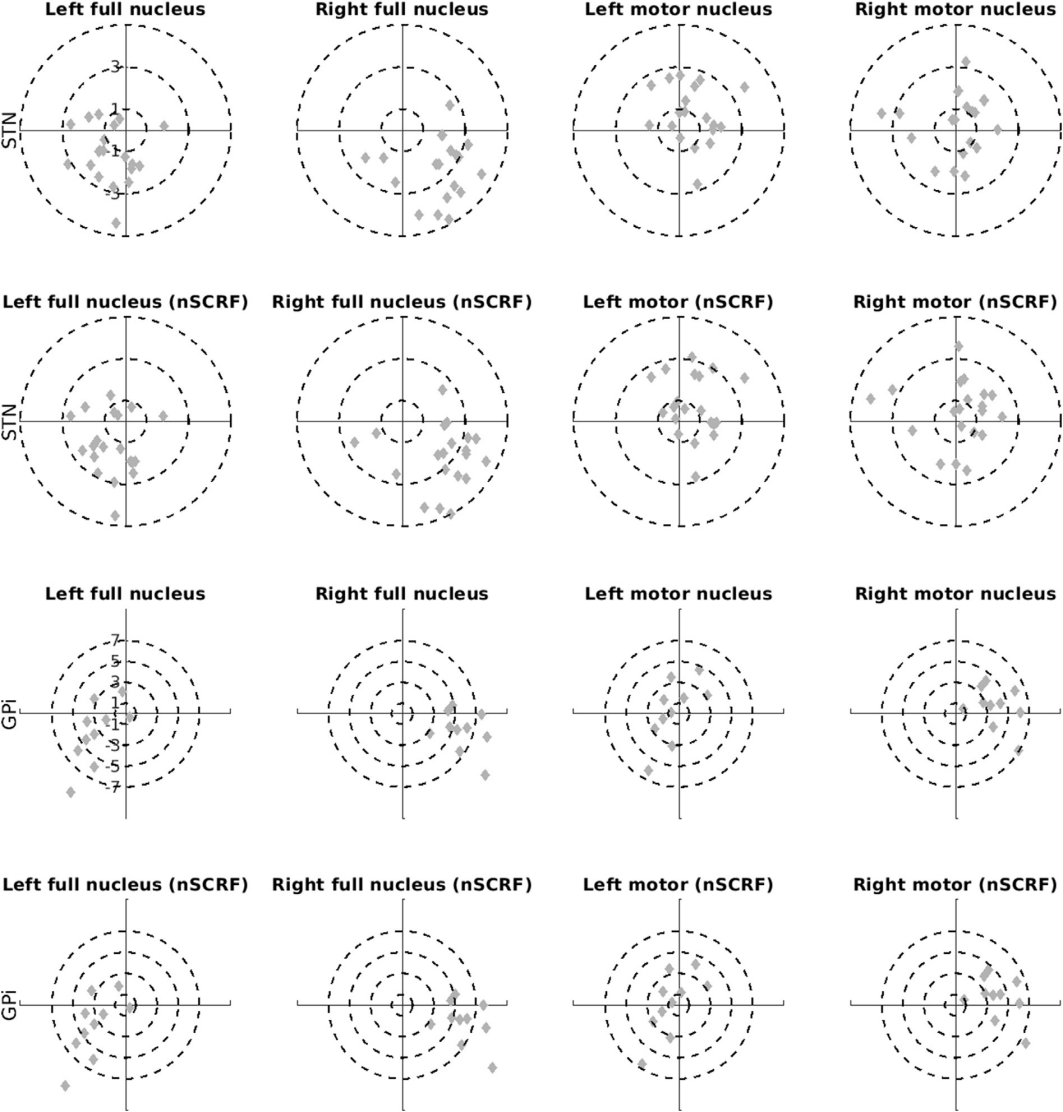
Target Plots of Electrode Location Electrode co-ordinates are displayed in relation to the target centre of gravity (see Fig. 1B left). This allows systematic visual inspection of lateral (X) and anterior (Y) plane variance viz a viz accuracy and precision of group targeting. These plots are systematically arranged per target (STN: upper two rows, GPi: lower two rows), nucleus (main: left two columns, motor: right two columns), and hemisphere. Rows two and four remove the subcortical refine (nSCRF) that compensates for brain shift from the corresponding co-ordinates in the row above.

Target plots of accuracy per nucleus, hemisphere, and target are presented in Fig. 5 to determine if variation in accuracy occurred predominantly in any single Cartesian (XYZ) dimension. Variance occurred predominantly in the X-dimension in the right hemisphere and was consistent across nucleus (STN versus GPi) and target (overall nucleus versus motor sub-nucleus) (Supplementary Fig. 4). Utilisation of subcortical refine methodology (as an adjustment for brain shift) did not affect accuracy in any single dimension.

### 3.6 Relationship of accuracy to pneumocephalus and brain shift

**Fig. 6:**
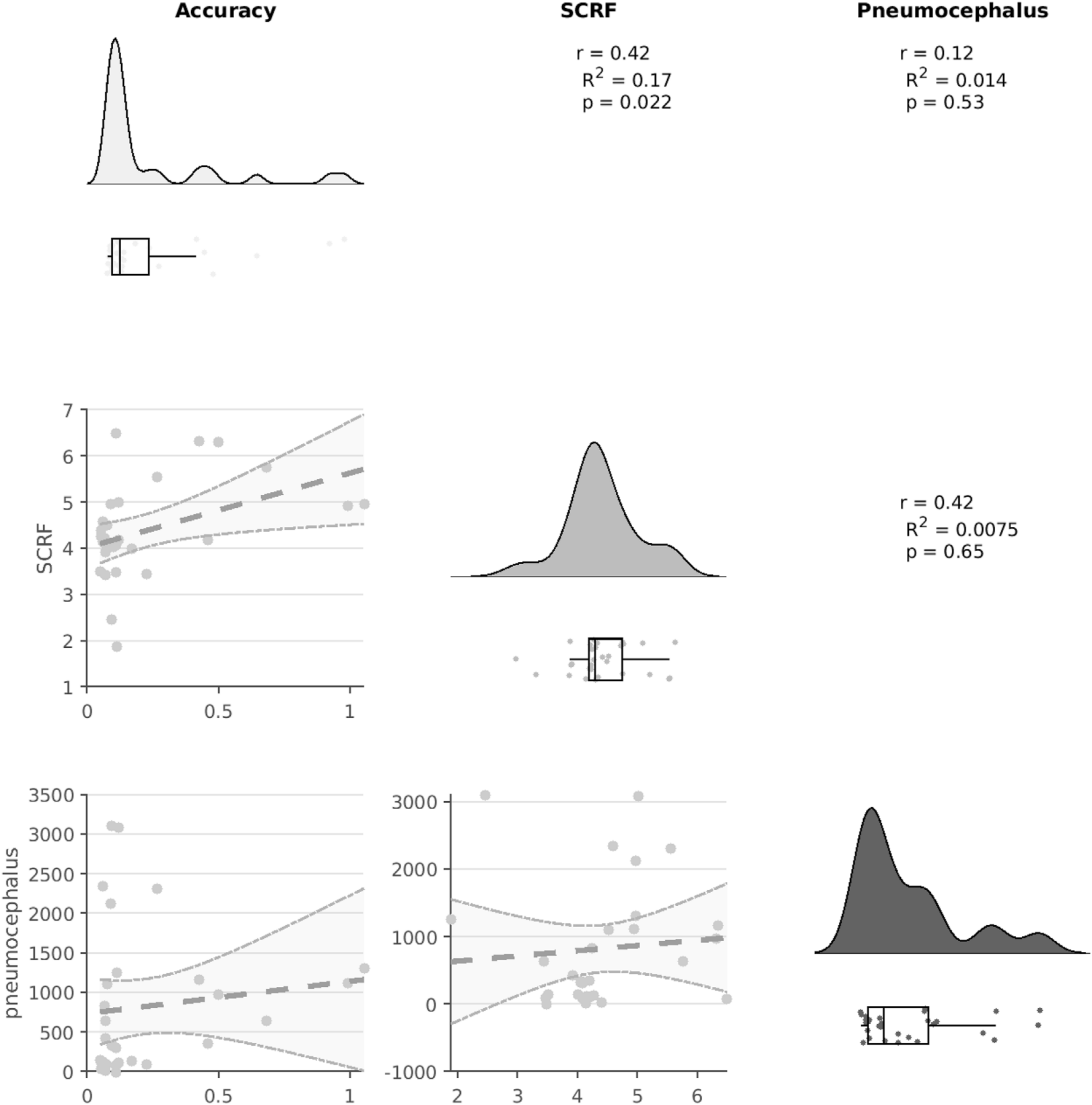
Accuracy, Pneumocephalus, and Brain Shift Electrode accuracy and averaged across hemispheres. Brain shift is reflected in the summed total of the subcortical refine (SCRF) transformation matrix. Pneumocephalus is assessed using a standard space brain mask (Fig. 1C). Increased brain shift, reflected in higher SCRF values, is correlated with reduced accuracy. However, accuracy is not correlated with brain shift, and brain shift is not correlated with pneumocephalus.

Analysis of factors that may impact upon accuracy, specifically pneumocephalus and brain shift, is presented in Fig. 6. Accuracy, in this instance defined as the mean accuracy across hemispheres, positively correlated with brain shift, as defined by the total adjustment performed by subcortical refine methodology, but this was not significant when corrected for multiple comparisons (r ​= ​0.42, R^2^ ​= ​0.17, p ​= ​0.022). There was no correlation between accuracy and pneumocephalus, nor between brain shift and pneumocephalus.

### 3.7 Accuracy and clinical outcomes

Finally, the relationship of accuracy to clinical outcomes is presented in Supplementary Figs. 5 and 6. Note that for GPi stimulation there were insufficient outcome data available for analysis and these data were excluded, hence these analyses are exclusively for STN stimulation. The VAT in either the main nucleus or motor sub-nucleus did not correlate with clinical outcomes. Furthermore, overall electrode accuracy did not correlate with the VAT in either the main nucleus or the motor nucleus (r ​= ​-0.19, p ​= ​0.43).

## 4 Discussion

In summary, we performed a systematic appraisal of DBS electrode accuracy using contemporary neuroimaging methods. Overall, accuracy was high. However, accuracy was lower in the GPi than STN, and for the second electrode implanted. This inaccuracy was found to occur predominantly in the X (lateral) dimension. Neither brain shift nor pneumocephalus were found to be associated with lower accuracy. Finally, electrode accuracy did not impact upon the total VAT able to be generated, nor on any one specific clinical outcome.

Lower accuracy in the second electrode implantation is a well-known issue. Risk factors include cerebral atrophy (Park et al., 2018). Recognised methods to address this include adding a specific offset to the final frame co-ordinates (Park et al., 2018) and performing staged surgery. It could also be argued that awake surgery with macrostimulation or microelectrode recordings, potentially with multiple tracts, would allow feedback of the ideal location. Other proposals include continuous irrigation after durotomy to minimise brain shift and pneumocephalus. Device hardware developments, for example local field potential recording of the best target or using directional stimulation, may facilitate compensation for lower accuracy. Nevertheless, with the lack of correspondence between accuracy and clinical outcomes or the VAT that was able to be generated, it would appear wise to not attempt elaborate methods of compensation for what in practice is apparently satisfactory accuracy.

Historically, accuracy has been appraised by comparing electrode implantations with that planned, typically using AC-PC co-ordinates. However, few consistent factors have emerged to guide improved accuracy. Furthermore, this method does not easily facilitate group analysis of multiple electrodes, comparison with functional templates (e.g. to delineate the motor subnucleus), or appraise systematic variance in targeting (i.e. electrodes could be precise and accurate but poorly planned within a nucleus). Our neuroimaging methodology addresses these shortcomings and allows a systematic appraisal of electrode inaccuracy accounting for both targeting and planning error. Using neuroimaging methods, we were able to not only identify the specific clinical situations where accuracy was lower, but appraise what factors were associated with accuracy. Our findings suggest that brain shift and pneumocephalus have less of an effect on accuracy that previously believed.

Nevertheless, a neuroimaging approach to accuracy should be seen as complimentary to rather than superseding traditional co-ordinate approaches. Strengths of the traditional co-ordinate approach include direct appraisal of how the final location compares to the intended target and comparison with individual rather than template-based anatomy. It also allows for a more clinically defined measure of accuracy in the order of millimetres from planned target, rather than the somewhat lower distances involved using our definitions of accuracy. However, when accuracy and clinical outlines are typically good, an extensive and detailed database of outcomes is necessary to identify subtle features that may be associated with accuracy in specific situations. For example, our sample size of n ​= ​38 would only be sufficient for detecting an r ​= ​0.45 with alpha ​= ​0.05 and beta ​= ​0.20 prior to multiple comparisons corrections (https://sample-size.net/correlation-sample-size). With this in mind, we have not appraised the effects of age or electrode type on accuracy, for example. Notably, our methods lend themselves to easily performing these subsequent analyses, and we have freely shared our code online to do so in the hope that other larger datasets will be able to test these hypotheses in the future.

Establishing a ground truth with which to verify the accuracy of electrode localisation is an ill posed problem without using post-mortem analysis(Reddy and Lozano, 2018). Limitations in electrode localisation include the difficulty in segmenting the STN automatically at the individual level, which even with 7 ​T MRI remains an evolving process(Visser et al., 2016). Group templates have been introduced to obviate this issue (as well as offering additional resolution and functional segmentation)(Visser et al., 2016). However, it is unclear how well they reflect individual anatomy, particularly in the context of a progressive neurodegenerative disease(Nowacki et al., 2018). Other limitations of image processing pipelines include those related to registration (which may be ameliorated to a degree by post-operative MRI(Zrinzo et al., 2016), and the utilisation of non-automated processes in electrode reconstruction (which is currently easier with post-operative CT and may also potentially allow directionality determination (Husch et al., 2018; Hellerbach et al., 2018). One must therefore be mindful that anatomical localisation data is only one aspect in considering optimal electrode targeting and must be considered alongside the complimentary neurophysiological and clinical parameters.

Overall, our accuracy was comparable with that presented in the literature, serving as a robust audit of our method (specifically, general anaesthesia throughout, frame-based, direct targeting, MRI-planned, and CT-verified)(Saint-Cyr et al., 2002; McClelland et al., 2005; Burchiel et al., 2013; Hamid et al., 2005; Bjartmarz and Rehncrona, 2007; Foltynie et al., 2011; Ko et al., 2017; Frizon et al., 2018)–(Saint-Cyr et al., 2002; McClelland et al., 2005; Burchiel et al., 2013; Hamid et al., 2005; Bjartmarz and Rehncrona, 2007; Foltynie et al., 2011; Ko et al., 2017; Frizon et al., 2018). This accuracy is juxtaposed with studies of microelectrode recordings have reported revision of the original imaging-based targeting in approximately 20%. Despite this emphasis on accuracy, in our series electrode localisation did not enable prediction of clinical outcomes, in contrast to that reported in the literature(Horn et al., 2019a, 2019b; Bot et al., 2018). One explanation for this could be a lack of statistical power related to sample size and data attrition. Furthermore, when using VAT analysis, stimulation parameters may not necessarily be optimal (due to either clinical factors or the multiple permutations of programming parameters)(Koeglsperger et al., 2019), which may obfuscate any relationship between accuracy and outcomes. Further work is required in exploring the relationship between clinical outcomes, electrode location, and determining what is clinically meaningful accuracy at the individual level.

Revision surgery has been documented as occurring in up to 15% (Frizon et al., 2018; Ellis et al., 2008; Falowski et al., 2015; Patel et al., 2015; Falowski and Bakay, 2016; Rolston et al., 2016; Nickl et al., 2019) with consequent effects on healthcare services, finances, and risk of surgical complications. Myriad technologies have been proposed with the aim of improving accuracy, including the use of robotics(Xu et al., 2018; Goia et al., 2019; Liu et al., 2019; VanSickle et al., 2019). In our data we identified three participants each with a single electrode location outside of the target nucleus by greater than 2 ​mm. Reassuringly, this did not lead to any adverse neurological outcomes or unsatisfactory treatment response – and therefore neither electrode was revised – but nor was there a clear clinical indication of why discrepancy from the usual accuracy occurred. Overall, these data suggest that the accuracy achieved in routine practice is sufficient to not adversely impact upon clinical outcomes.

Strengths of our study include the implementation of a relatively lightweight design and analysis strategy that integrates efficiently with a busy clinical movement disorders practice. Furthermore, we have released detailed open-source code to make this process more accessible. Limitations include the overall numbers and attrition, although these data represent a realistic reflection of what can be achieved in routine clinical practice. Improvements include focusing on streamlining data collection and optimising imaging parameters. However, the main factor that will play into improving study power will be the establishment of multi-centre collaborations and open-source datasets.

Emergence of open-source lead localisation software compliments an overall burgeoning in DBS hardware and research. Accurate appraisal of lead localisation is not only useful in deep brain stimulation surgery, but also in lesioning, cell delivery studies, and stereo-electroencephalography (SEEG). Furthermore, lead localisation can be used to appraise changes to clinical practice (such as a change in imaging sequences, surgical workflow, or head position), which can now be objectively audited. This work therefore represents an ideal platform for large multi-centre audits and specifically trainee projects(Chari et al., 2017). Such research, when performed systematically and with sufficient statistical power, may go some way to improving our understanding of accuracy and precision, as well as deriving optimal surgical workflows.

## 5 Conclusions

In conclusion, our analysis is supportive of the accuracy in performing deep brain stimulation in a fully image-guided manner under general anaesthesia, but highlights the complexity of understanding accuracy, and cautions about lower accuracy during the second electrode. We hope that publication of these data and resources will encourage groups to utilise developments in electrode localisation, develop collaborations, and provide large open-source datasets that enhance our understanding of outcomes after deep brain stimulation.

## Declaration of competing interest

The authors declare that they have no known competing financial interests or personal relationships that could have appeared to influence the work reported in this paper.

## Figures and Tables

**Multimedia component 2:**
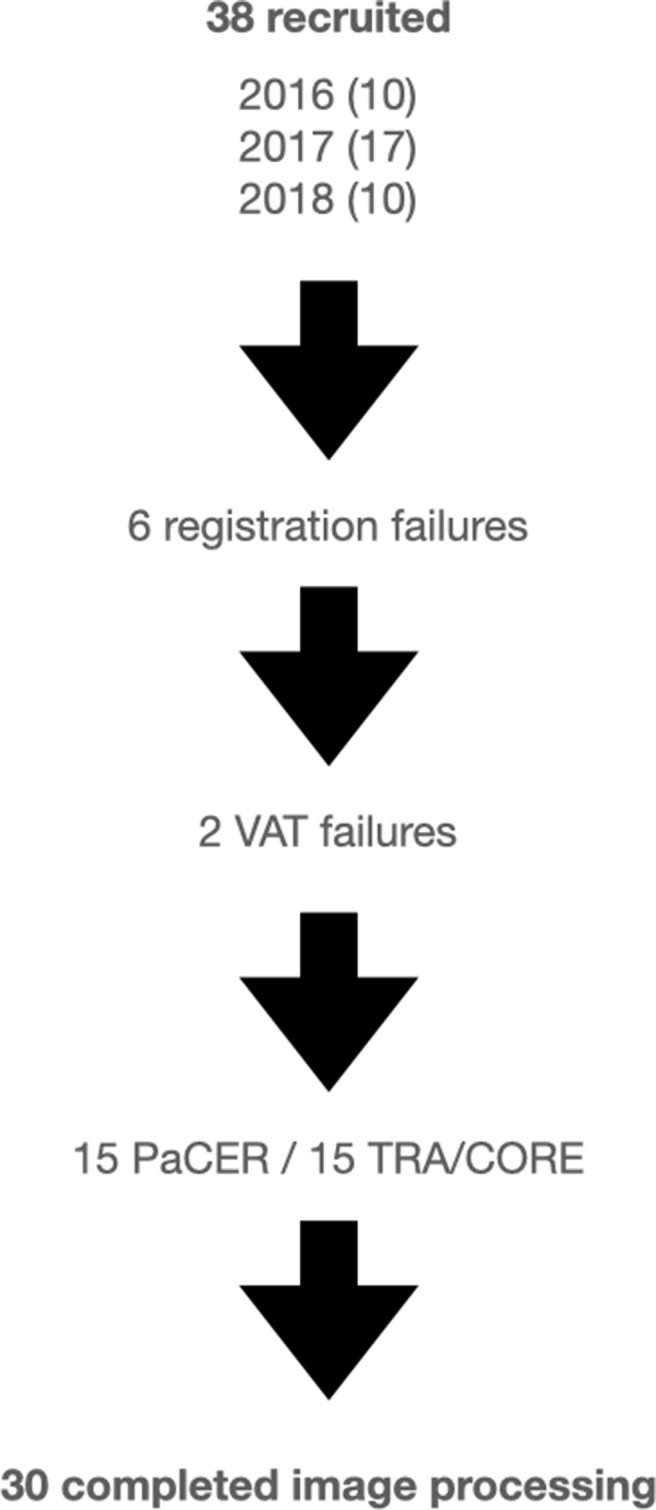


**Multimedia component 3:**
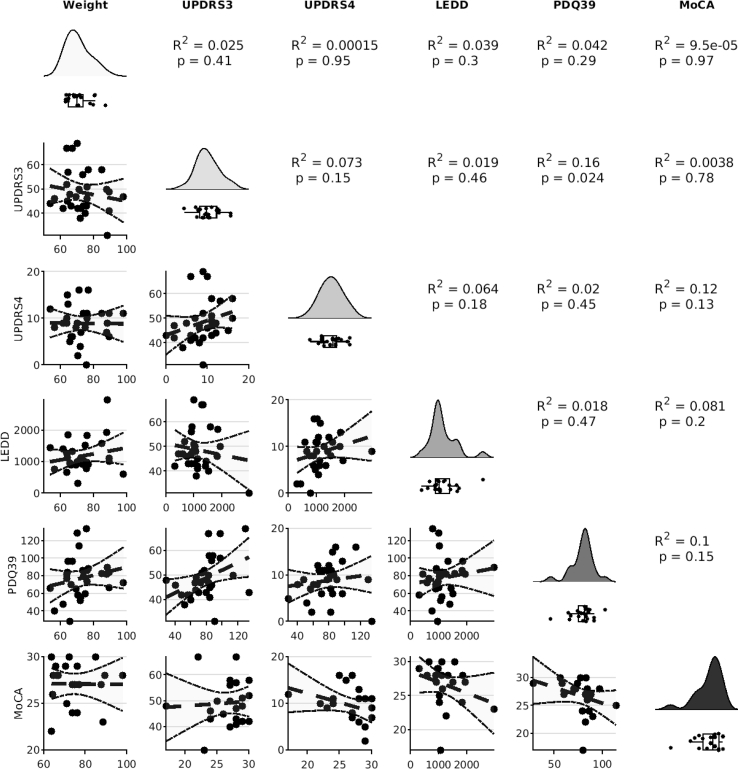


**Multimedia component 4:**
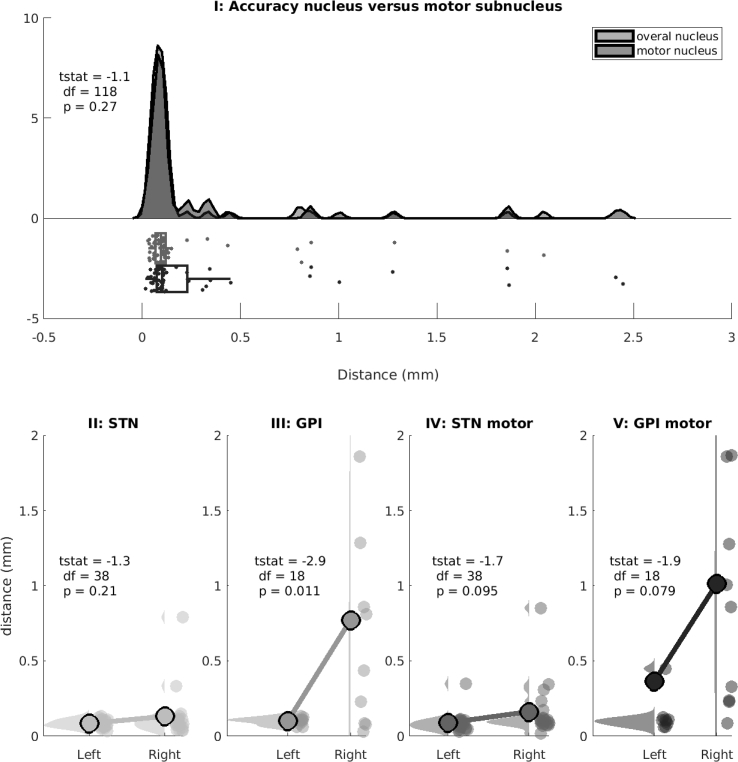


**Multimedia component 5:**
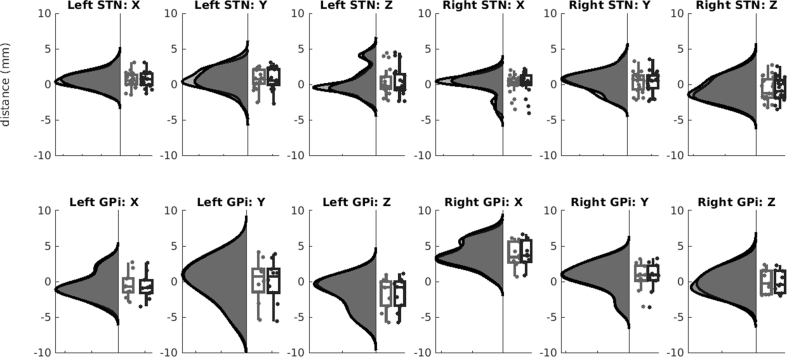


**Multimedia component 6:**
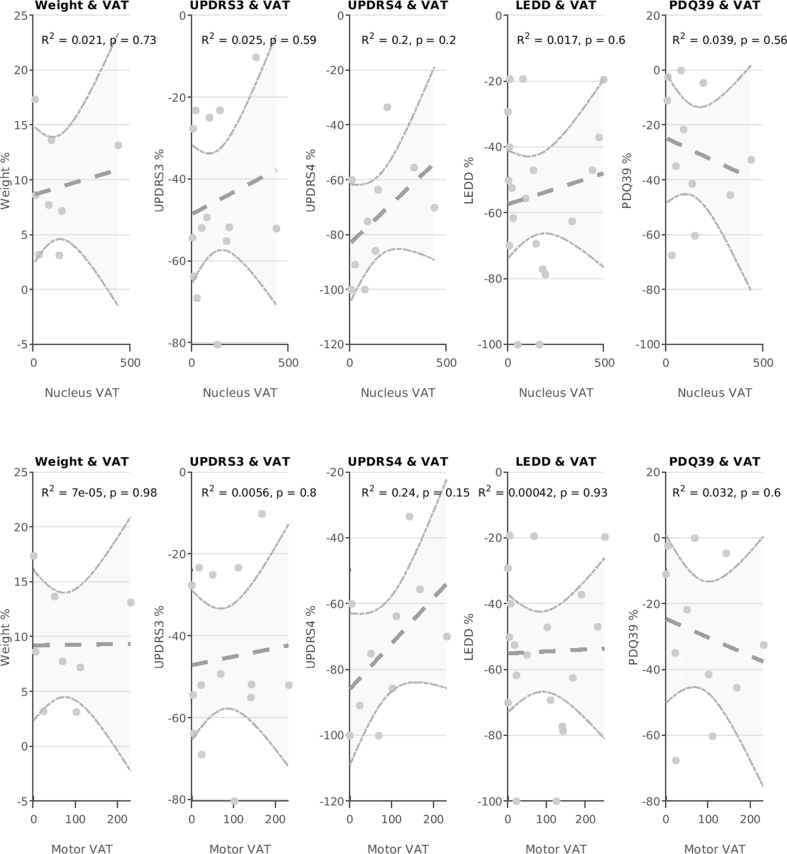


**Multimedia component 7:**